# Impact of 2019 Novel Coronavirus (2019-nCov) Pandemic and Lockdown on Parents and Caregivers in Ontario, Canada

**DOI:** 10.1177/00099228231155004

**Published:** 2023-03-01

**Authors:** Shaneela Shahid, Janet Weisz, Ivan D. Florez

**Affiliations:** 1 Department of Health Research Methods, Evidence, and Impact, McMaster University, Hamilton, ON, Canada; 2 Department of Pediatrics, McMaster Children’s Hospital, McMaster University, Hamilton, ON, Canada; 3 Department of Pediatrics, University of Antioquia, Medellin, Colombia; 4 School of Rehabilitation Science, McMaster University, Hamilton, ON, Canada

**Keywords:** COVID-19, pandemic, parents, well-being, children, anxiety

## Abstract

The COVID-19 pandemic has impacted parents’ and children’s well-being. This study aimed to evaluate the impact of the COVID-19 pandemic and its preventive measures on children’s well-being and their parents’ anxiety level. Parents/caregivers were invited to respond to a self-administered survey. The primary outcome was to assess the rate and severity of parental anxiety during the pandemic/lockdown. Four hundred and thirty parents completed the survey. Ninety-two (21%) and 10 (2%) parents reported that their children gained or lost weight during the pandemic, respectively. Eighty-one (19%) parents reported a regression in their children’s developmental milestones, particularly in toileting, speech, and social interaction. The GAD-7 mean scores increased by 2.9 points (95% CI [2.5, 3.25]; *P* < .001) in comparison with prepandemic scores. Adjusted multivariable analysis showed that having children with psychological conditions and a maternal education level less than a university degree were significantly associated with higher parental anxiety.

## Background

The COVID-19 pandemic was declared by the World Health Organization on March 12, 2020. Various preventive measures such as lockdown restrictions, social and physical distancing, quarantine, and self-isolation have been implemented to reduce the spread of COVID-19. Lockdown (confinement) is one of the most restrictive measures commonly imposed by governmental authorities globally. Some adverse psychological effects of confinement in adults, such as anxiety, depression, stress, and post-traumatic stress symptoms, among others, have been described.^1^

In June 2020, Statistics Canada surveyed the impact of COVID-19 on Canadians parenting during the pandemic. Parents reported their concerns about balancing work and childcare, social isolation, increase in screen time, and less physical activity; however, this survey did not inform us about the impact of the pandemic on parents’ mental well-being, particularly anxiety children’s well-being including mental health, developmental milestones, physical activity, and sleep.^2^ Moreover, it did not report how the public health measures, such as lockdown restrictions, had impacted parents’ and children’s well-being.^2^ Furthermore, the Statistics Canada survey was conducted earlier in the pandemic, and as such, the extent of the impact of the pandemic and the lockdown restrictions were not captured. In Ontario, a state of emergency was declared on March 17. Lockdown restrictions were put in place on March 30, which were extended multiple times till July 29. Schools in Ontario remain closed from mid-march 2020 till the end of the school year. The impact of these measures has not been measured.

Therefore, we surveyed Ontario’s parents and caregivers to describe the parental understanding and attitude toward preventive measures, parental emotional well-being, and parental preferences for sending the child back to daycare/school.

## Material and Methods

This cross-sectional study was conducted in southern Ontario, Canada, from October to December 2020. We developed a questionnaire comprising 80 multiple-choice questions piloted with some parents broadly disseminated. The survey was anonymous, and it took approximately 10 minutes to complete. We used a convenience sampling method to recruit the participants. Parents/caregivers were invited to participate via phone or during their visit to the physician’s office of 2 researchers (SS and JW).

Our primary outcome was the percentage of anxiety in parents/caregivers during the pandemic, assessed using the Generalized Anxiety Disorder-7 (GAD-7) tool. The GAD-7 is a self-rating reliable and validated tool with 7 items used to measure anxiety in the general population.^3,4^ To compare the parental level of anxiety before and during the pandemic, in the same survey, we requested parents to recall the level of anxiety before the pandemic using the GAD-7 scale. After capturing the baseline characteristics of participants, the GAD-7 scale was introduced to assess the prepandemic parental anxiety level. For the assessment of the parental anxiety level during the pandemic, the GAD-7 was re-introduced in the later part of the survey. We applied the tool to measure parental anxiety before and during the pandemic. The total score ranges from 0 to 21, and a score of 10 or greater is considered a reasonable threshold to identify anxiety cases.^3^ We categorized minimal to mild scores as low levels of anxiety and moderate to severe scores as a high level of anxiety. Secondary outcomes included the children’s well-being measured with mean screen time, regression in development, weight change, and hours of sleep before and during the pandemic.

Descriptive statistics were used to present parents/caregivers and their children’s characteristics, which included age, gender, number of siblings, education, working position, and parental anxiety, as well as a parental understanding of lockdown and its impact on child and parent feelings/emotions, sleep, screen time, developmental regression and extracurricular activities by comparing these variables before and during the pandemic.

A bivariate analysis was performed to explore variables (characteristics of the parents/caregivers) associated with both children’s and caregivers’ well-being, particularly parental anxiety. Quantitative variables are presented as mean and standard deviations (SDs) or median and interquartile range (IQR), according to their distribution, while qualitative variables as frequencies and proportions. The chi-square test was performed for categorical variables, and the *t*-test for continuous variables. A *P*-value of <.05 was considered statistically significant.

A multivariable linear regression model explored factors predicting parental anxiety levels. These were introduced sequentially into the model to predict the primary outcome. These predictors/factors were chosen based on evidence from similar studies on the COVID-19 pandemic^5^ and their clinical importance. Data analysis was performed using IBM SPSS version 27 and Microsoft Excel 2019 edition.

The sample size calculation supported the need to identify the prevalence of anxiety, our primary outcome. A study in the United Arab Emirates found a pandemic-related prevalence of parental anxiety of 71%.^6^ Based on this expected prevalence, at least 400 participants were needed with a confidence interval of 95% (95% CI) and a precision of 5%.^7^ Since the response rate of surveys is around 30% to 40%, we invited 1000 parents/caregivers to obtain 400 responses. The Hamilton Integrated Research Ethics Board approved this study in September 2020 (ID#12571).

## Results

A total of 1000 parents/caregivers were invited, of which 712 agreed to participate, and 430 (response rate, 43%) completed the survey and were included. Respondents were completed the survey and were included. Respondents were mostly female, and the mean age was 38.6 (SD = 6.0) years (Table 1). Most of these parents had college/university degrees. More than 50% of parents had 2 or more children at home. Fifty-four families (13%, 95% CI [9.5%, 16%]) and 113 (26%, 95% CI [22%, 30%]) reported having at least 1 child with medical or psychological health conditions, respectively. Common psychological health conditions in children include attention-deficit/hyperactivity disorder (ADHD), comorbid anxiety, and autism spectrum disorder (ASD) (Table 1).

**Table 1.** Participants Characteristics.

Characteristics	N (%)
Gender of parent	
Female	376 (87%)
Male	54 (13%)
Age of parent (mean ± SD)	38.6 (6.0) years
Marital status	
Common law	28 (6.5%)
Married	372 (86.5%)
Separated/divorced	17 (4%)
Single parent	13 (3%)
Maternal education	
Elementary school	2 (1%)
High school/vocational school	25 (5%)
Some college	124 (29%)
University degree	279 (69%)
Father education	
Elementary school	3 (1%)
High school/vocational school	60 (14%)
Some college	124 (29%)
University degree	243 (56%)
Number of children at home	
One child	24%
Two or more children	66%
Children with physical problem	54 (13%)
Type of physical problem—asthma/allergies	16 (30%)
Children with psychological problem	113 (26%)
Type of psychological problem	
ADHD and comorbid	40 (35%)
Anxiety	36 (32%)
ASD	8 (7%)
Others	29 (26%)
Lockdown restriction	390 (91%)
Duration of lockdown	
Less than 1 month	1 (1%)
1-2 months	19 (4.4%)
3-4 months	56 (13%)
5-6 months	83 (19.3%)
More than 6 months	231 (54%)
Quarantine	42 (10%)
Duration of quarantine	
1-2 weeks	28 (67%)
3-4 weeks	9 (21%)
4-8 weeks	4 (10%)
8-12 weeks	1 (2%)

Most of the participants were female parents and most of these parents had either college or university degrees. More than 50% of households had 2 or more children at home and one-fourth of these parents reported having children with psychological conditions.

Abbreviations: SD, standard deviation; ADHD, attention-deficit/hyperactivity disorder; ASD, autism spectrum disorder.

### Pandemic and Lockdown Restrictions

Three hundred and ninety (91%, 95% CI [87%, 93%]) parents reported following the lockdown restrictions. Four hundred and ten participants (92%, 95% CI [93%, 97%]) followed the lockdown restriction for 3 or more months. Three hundred and eighty-three parents (90%, 95% CI [86%, 92%]) reported that staying at home protects everyone, including the community. Only 42 (8.9%, 95% CI [8%, 9.1%]) respondents had to quarantine due to exposure to COVID-19 cases.

During pandemic and lockdown restrictions, parents reported more than one of the following feelings: sad, helpless, anxious, isolated, frustrated, lonely, confused, restless, angry, annoyed, overwhelmed, and loss of control. However, feelings of being happy, peaceful, and grateful were also reported by the parents. The pandemic impact on the parents’ working situations was minimal, where more than 50% of parents continued to work either remotely or commute to work, and 19 (4%, 95% CI [3%, 7%]) mothers and 13 (3%, 95 CI [1%, 5%]) fathers reported that they were either laid off/loss of work or not working due to pandemic.

### Impact on Parents

**Figure 1.**
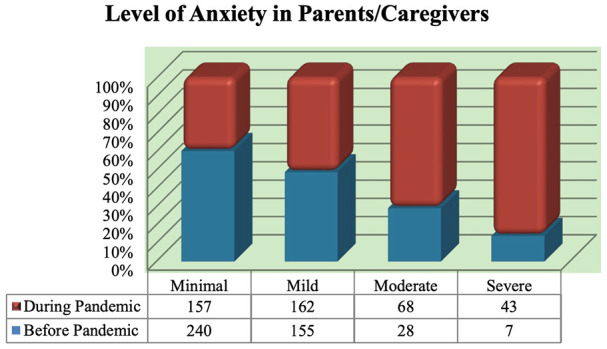
Level of anxiety in parents/caregivers before and during the pandemic. The level of anxiety has increased in parents during the pandemic and there was significant proportion of the parents experiencing moderate to severe anxiety.

Most respondents reported anxiety around parenting (241; 56%, 95% CI, [51%, 60%]) higher in female parents (88% vs 12%). Before the pandemic, parents reported their anxiety as minimal to mild; however, anxiety increased (Figure 1). Respondents also reported the positive impact of the COVID-19 pandemic and lockdown restrictions, such as spending more time with family, exploring indoor activities, practicing better hygiene, and connecting with other family members remotely.

Table 2 shows the bivariate analysis of parents/children characteristics and level of anxiety in parents during the pandemic. Having children with psychological health conditions was associated with a high level of anxiety in parents during the pandemic. Also, highly anxious parents had children who were more likely to feel anxious than non-anxious parents (χ^2^ = 61.898; *P* < .001). This survey shows an increment of anxiety mean score on the GAD-7 scale by 2.9 points (95% CI [2.5, 3.25]; *P* < .001) during the pandemic compared with the prepandemic period. Multivariable analysis showed that having children with psychological conditions and maternal education level less than a university degree were significantly associated with higher parental anxiety during the pandemic when adjusted for the age, gender of the parent, duration of the lockdown, and the number of children at home (Table 3).

**Table 2.** Bivariate Analysis—Parental/Children Characteristics and Level of Anxiety in Parents (GAD-7) During the Pandemic.

Parents/child(ren) characteristics	Low level of anxiety in parents N (%)	High level of anxiety in parents N (%)	*P*-value
Age of parents			χ^2^ = 2.0 (*P* = .150)
≤30 years of age	21	12	
>30 years of age	298	99	
Gender			χ^2^ = 0.318 (*P* = .573)
Female	278	99	
Male	41	111	
Maternal education			χ^2^ = 0.22 (*P* = .641)
Elementary school/high school and college	110	41	
University	209	70	
Number of children at home			χ^2^ = 0.114 (*P* < .736)
2 or less children at home	248	88	
3 or more children at home	71	23	
Children with physical health condition			χ^2^ = 2.83 (*P* = .092)
Yes	35	19	
No	284	92	
Children with psychological condition			χ^2^ = 6.057 (*P* = .014)
Yes	74	39	
No	245	72	
Duration of lockdown			χ^2^ = 3.576 (*P* = .167)
≤6 months	151	47	
>6 months	168	63	
Family member with medical condition or over 60 years of age			χ^2^ = 0.526 (*P* = .468)
Yes	78	31	
No	241	80	
Child feeling anxious during the pandemic			χ^2^ = 61.898 (*P* < .001)
Yes	131	63	
No	188	48	

This study shows that parents had experienced high level of anxiety if they have children with psychological health conditions. *P*-value = Using χ^2^ (chi-square) test.

**Table 3.** Multivariable Regression Model for Study Variables to Predict Anxiety in Parents During the Pandemic.

Variables	Coefficient B	SE	β	95% CI	*t*	*P*-value
Intercept	8.983	2.190		4.67, 13.28	4.101	.000
Age	−0.069	0.044	−.079	−0.156, 0.018	−1.561	.119
Gender	−0.306	0.763	−.019	−1.806, 1.194	−0.401	.689
Children with psychological conditions	1.921	0.601	.161	0.740, 3.102	3.197	**.001**
Maternal education	−1.109	0.537	−.101	−2.164, –0.053	−2.064	**.040**
Duration of lockdown	0.576	0.498	.055	−0.403, 1.554	1.157	.248
No. of children at home	−0.244	0.324	−.037	−0.881, 0.393	−0.752	.452

Multivariable analysis showed that having children with psychological health conditions and maternal education level less than university were significantly associated with higher parental anxiety during the pandemic. Bold values indicate statistical significance at the *p*<0.05 level. *P*-value = Multivariable linear regression.

Abbreviations: SE, standard error; CI, confidence interval.

### Impact on Children

**Figure 2.**
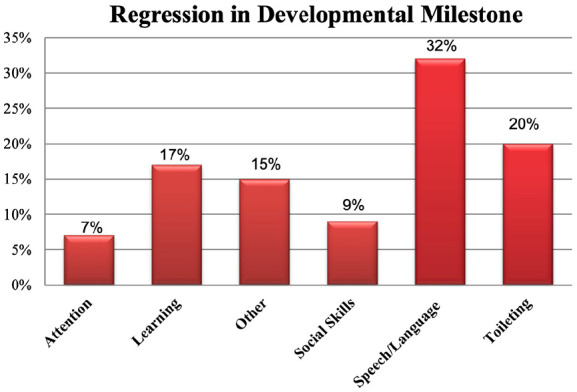
Regression in developmental milestones. During the pandemic, there was regression in developmental milestones in children particularly in speech/language, toileting and learning.

Ninety-two (21%, 95% CI [17%, 25%]) and 10 (2%, 95% CI [1.7%, 2.5%]) parents reported that their children gained or lost weight during the pandemic, respectively. Furthermore, 80 (19%, 95% CI [15%, 22%]) parents reported a regression in their children’s developmental milestones, particularly in speech/language, toileting, learning, and social interaction (Figure 2). During the pandemic, children spent more time on screen (1.9 vs 3.7 hours) with a mean increment of screen time of 1.8 hours (95% CI [1.6, 1.9]; *P* < .001). There was no change in the number of hours of sleep during the pandemic compared with the prepandemic. Most parents reported that their children had started to attend in-person school since September 2020 (75%, 95% CI [70%, 79%]). Only 13 (3%, 95% CI [1%, 6%]) parents planned to switch from in-person to online learning due to the pandemic. Some of the positive changes parents had noted since their children started school included appearing happier and physically active, improving their mental well-being and academic performance, sleeping better and less time on technology.

### Adhering to Public Health Measures and Seeking Medical Advice

**Figure 3.**
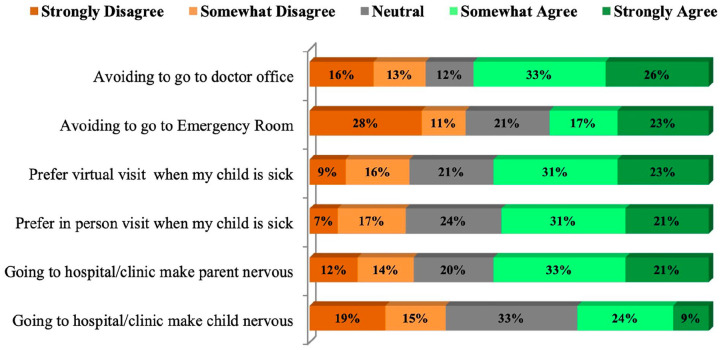
Level of agreement using Likert scale. During the pandemic, parents agreed to avoid the doctor’s office or emergency room (ER) visit when their child(ren) unwell as it was making them more anxious and preferred to be seen virtually.

Four hundred and twenty (97%, 95% CI [95%, 98%]) parents reported using masks and use of sanitizer to disinfect their hands during their visit to the doctor’s office or emergency room (ER) visit. More than 50% of parents agreed to avoid going to the doctor’s office (59%, 95% CI [51.4%, 68.1%]) and prefer to see the doctor virtually (54%, 95% CI [47.4%, 63.3%]). More than 50% of parents agreed to go to the hospital, and the doctor’s office makes them anxious (53%, 95% CI [45%, 61%]) (Figure 3). One-fourth (20%, 95% CI [16%, 24%]) of the parents reported that their child was unwell and did not go ER or to the doctor’s office as they were anxious.

## Discussion

This study has highlighted the significant impact of the COVID-19 pandemic and lockdown restrictions on parents’ and children’s well-being. Based on our best knowledge, this is the first cross-sectional study reported on the impact of the COVID-19 pandemic on parents’ and children’s well-being at the community level in Ontario. Most participants reported worsening anxiety levels during the pandemic, mainly if they had children with previously diagnosed psychological conditions. Participants also reported the impact of pandemic and lockdown restrictions on children’s well-being, including regression of milestones, weight gain, and an increment in screen time, and feeling more anxious, particularly if their parents are experiencing anxiety.

Our results concur with other studies on parents during the COVID-19 pandemic.^5-9^ A national survey from the United States reported one-third of parents reported worsening their mental health during the pandemic compared with the prepandemic period. Still, they did not describe the impact on parents.^10^ Possible explanations for worsening anxiety are social isolation, balancing work and child care, and increased care demand from children. Moreover, it was noted that having children with psychological needs is a significant predictor of parents’ anxiety, and similar results are reported elsewhere.^5^,^11-13^ Moreover, mothers with lower education levels had higher anxiety levels.^14^

Parents reported a perceived weight gain in their children during the pandemic in approximately one-fourth of the cases. The relationship between lockdown and weight gain has been previously described.^15-17^ The factors that may play a role in weight gain are low physical activity, increased screen time, unhealthy food choices, and sugary food availability.^18-20^ Although we did not study the risk factors for weight gain, it is believed that the same factors may have played a role in our cohort. Only 1 small study reported objective weight gain in children, which found this increase by 25% to 50%.^20^

Based on our knowledge, this is the first Canadian study highlighting the impact of lockdown and the pandemic on children’s developmental milestones, particularly in speech/language, toileting, and learning. Regression in developmental milestones has been suspected in children during stressful times. This has been a significant concern for clinicians and caregivers during the pandemic. A retrospective analysis of the 2003 SARS pandemic showed that children had delayed childhood development, including gross motor and language development.^21^ Given our study design, it is impossible to confirm and quantify the extent of the regression in our children’s developmental milestones. Further research needs to be designed to confirm these findings.

As clinicians, we always have concerns about screen time in children due to its potential impact on development and behavior.^22^ During the lockdown, there were limited outdoor activities at home, and parents had to work remotely and balance child care and work. Screen time is one of the ways which distract the child and preoccupy them during the daytime. Several authors have raised their concerns during the COVID-19 pandemic and have reported increased screen time in children.^23,24^ We found that before the pandemic, the average screen time for children was approximately 2 hours within the limit of the recommended screen time.^25^ Far worse, this screen time doubled during the pandemic. Nonetheless, we have limited information on how children use this screen time. We cannot be sure whether this time was used for creative, educational, and socializing activities, positively impacting child development.^26^

Our study is unique as we also identified the positive impact of the COVID-19 pandemic and lockdown. We found increased family bonding, hygiene measures, indoor activities, and children’s connection with other family members. We could not find much external evidence on this aspect, except a qualitative study highlighting how families could spend more time with their children, and parents were more attentive to their children’s well-being and happiness.^27^

We found that approximately one-fifth of respondents did not go to the ER or the doctor’s office when their children were unwell. The anxiety and the fear of getting infected may have prevented some parents from visiting doctors/ER. Further studies to evaluate the impact of this reduction in ER and doctor’s office visits, for instance, determining whether children may have had delays in treatments or diagnostics, are warranted.

There are many strengths of our study. We had a relatively high recruitment rate that exceeded our sample size. We performed a regression analysis to explore the predictors associated with worsening anxiety in parents. Also, we performed an objective assessment of anxiety using a validated tool to rule out generalized anxiety levels. Finally, this is the first study in Canada to report essential findings on regression in children’s developmental milestones due to pandemic and lockdown.

However, there are some limitations to mention. We used a convenience sampling method, and this survey was not open to the public. It is believed that participants who did not have the time or internet availability could not submit their responses. There are concerns related to selection bias and the generalizability of the findings. There is also concern about recall bias in reporting parental anxiety in the prepandemic period, as information about parental anxiety before the pandemic was also gathered in this survey from the participants.

In conclusion, the COVID-19 pandemic has significantly impacted both parents and children. The level of anxiety has increased in parents during the pandemic, particularly if they have children with psychological health conditions. We found an alarming increase in screen time, weight gain, and regression in developmental milestones in children. There is a need for future studies to understand the psychological impact of the pandemic and lockdown on parents and children. We need to develop effective screening and coping strategies for psychological conditions in parents and children due to the pandemic and lockdown restrictions; moreover, we need to formulate interventions that improve parents’ and their children’s physical and mental well-being.

## Author Contributions

SS: Participated in the study concept and design: contributed to acquisition, linkage, analysis, and interpretation of data, and drafting and reviewing the manuscript and approved the final version. IDF: Participated in the study design; contributed to data acquisition, linkage, analysis, and interpretation; drafted and reviewed the manuscript; and approved the final version. JW: Contributed to study design, data analysis interpretation, and manuscript review; and approved the final version.
